# Response to the COVID-19 Pandemic in Classrooms at the University of the Basque Country through a User-Informed Natural Ventilation Demonstrator

**DOI:** 10.3390/ijerph192114560

**Published:** 2022-11-06

**Authors:** Iñigo Rodríguez-Vidal, Alexander Martín-Garín, Francisco González-Quintial, José Miguel Rico-Martínez, Rufino J. Hernández-Minguillón, Jorge Otaegi

**Affiliations:** CAVIAR Research Group, Department of Architecture, University of the Basque Country UPV/EHU, Plaza Oñati, 2, 20018 Donostia-San Sebastián, Spain

**Keywords:** indoor air quality (IAQ), COVID-19, natural ventilation, schools, airborne infection risk, thermal comfort, human-building interaction, monitoring, retrospective analysis

## Abstract

The COVID-19 pandemic has generated a renewed interest in indoor air quality to limit viral spread. In the case of educational spaces, due to the high concentration of people and the fact that most of the existing buildings do not have any mechanical ventilation system, the different administrations have established natural ventilation protocols to guarantee an air quality that reduces risk of contagion by the SARS-CoV-2 virus after the return to the classrooms. Many of the initial protocols established a ventilation pattern that opted for continuous or intermittent ventilation to varying degrees of intensity. This study, carried out on a university campus in Spain, analyses the performance of natural ventilation activated through the information provided by monitoring and visualisation of real-time data. In order to carry out this analysis, a experiment was set up where a preliminary study of ventilation without providing information to the users was carried out, which was then compared with the result of providing live feedback to the occupants of two classrooms and an administration office in different periods of 2020, 2021 and 2022. In the administration office, a CO_2_-concentration-based method was applied retrospectively to assess the risk of airborne infection. This experience has served as a basis to establish a route for user-informed improvement of air quality in educational spaces in general through low-cost systems that allow a rational use of natural ventilation while helping maintain an adequate compromise between IAQ, comfort and energy consumption, without having to resort to mechanical ventilation systems.

## 1. Introduction

Most of the educational buildings in Spain rely solely on window opening as the means of ventilation throughout the whole school year. This makes it very difficult to control indoor air quality (IAQ) or even effectively know the state of ventilation in classrooms and educational spaces in general, as manual operation of natural ventilation is influenced not only by external factors (thermal comfort, noise, pollution), but also by the teaching requirements (e.g., room darkening) and ultimately the preferences of teachers, professors and students. 

From the energy crisis perspective, uncontrolled natural ventilation involves very substantial energy losses that could be prevented [1,2], while thoroughly planned, adequate natural or hybrid ventilation can be an effective means of saving energy [3,4,5]. On the other hand, from the teaching policy perspective, poor indoor air quality is widely accepted to negatively impact students’ academic performance [6,7]. Regarding the public health standpoint, insufficient ventilation and some of its common consequences have associated health effects [8,9,10]. Both health and academic performance are intertwined, as studies showed less healthy classrooms reduced school attendance [11,12]. In the context of the reopening of face-to-face classrooms in September of 2020 [13], when it was already understood that airborne transmission of SARS-CoV-2 was the pre-eminent driving force of the COVID-19 pandemic, obtaining information that could help to improve IAQ and reduce contagions was a priority in all schools and educational facilities. 

With the resources available to the Campus Bizia Lab research initiative [14] and with the intent to use the university’s own facilities as living laboratories, a pilot experiment was developed and deployed in the School of Architecture of the University of the Basque Country, which was presented in the literature [15,16]. 

Several studies analysed the effect of user information systems focused on analysing their effect on Indoor Air Quality [17,18,19,20,21]. During and after the pandemic, some studies [22,23,24] analysed the effect of the new patterns that impacted users through the ventilation protocols implemented in institutions and buildings. In this regard, this study delves into the new environmental conditions generated in indoor educational spaces as a consequence of the ventilation protocols implemented and also presents an additional case study. To this end, the methodology was based on three clearly differentiated phases that allowed us to analyse user behaviour patterns as well as their effect on the IAQ of these spaces. In this way, this study made available the results obtained on the IAQ so that future measures in other case studies can be based on the experience acquired by the UPV/EHU experiment.

The objectives of the UPV/EHU experiment were to assess the effectiveness of natural ventilation in the studied spaces, provide real-time information on indoor air quality and, ultimately, to position the user of university spaces as an active player in improving the health of their classroom or workplace and to provoke learning around IAQ issues supported by the real data collected by the installed monitoring systems. 

### 1.1. Ventilation in Educational Buildings in Europe, Spain and the Basque Country

In Europe, 17% of nonresidential buildings are for educational purposes. According to the Cadaster census in Spain (excluding the Basque Country and Navarre), there are 51,349 cultural buildings [25], among which are mostly buildings used for education. In the Basque Country there are 1209 nonuniversity schools (600 in Bizkaia, 426 in Gipuzkoa and 183 in Alava) with 17,470 classrooms in total [26]. As for the university field, the University of the Basque Country alone has 32 Schools and Faculties distributed in three campuses throughout the Basque territory. 

The Spanish regulation on indoor air quality and ventilation for educational spaces is the *Reglamento de Instalaciones Térmicas de los Edificios* (RITE) [27], whose first document dates back to 2007 [28]. Between 90% and 95% of educational buildings were built before the aforementioned regulation, which promoted effective requirements for ventilation, air quality and indoor comfort [29]. Some 60% of these centers were created before any related regulations existed, namely Royal Decrees 1618/1980 [30] and 2429/1979 [31], which approved the first heating and cooling standard and the well-known NBE-CT-79.

In 2014 the Schools Indoor Pollution and Health Observatory Network in Europe [32] performed a study in 337 classrooms across Europe with 112 schools in 23 countries, which obtained the following relevant data:

- 81% of the studied samples ventilated just by means of opening and closing windows.
- Out of the buildings with mechanical ventilation systems, only 47% were equipped with an air quality control system that used CO_2_ concentration as a metric.
- 88% of the classrooms performed some ventilation action during breaks between lessons, while 70% kept the windows opened during classes also.

The results of the 300 elementary classrooms analysed in the study, which set an air quality benchmark of 1000 parts per million (ppm) of CO_2_, showed a mean value of 1433 ppm, with a median of 1257 ppm and minimum and maximum values of 269 and 4960 ppm, respectively, with a standard deviation of 856 ppm. In 25 nurseries the results obtained were 1309 ppm (mean), 1065 ppm (median) and a minimum and maximum value of 394 and 3530 ppm, respectively, with a standard deviation of 892 ppm. In both cases a mean ventilation rate of one air change per hour was estimated.

Numerous previous works showed that natural ventilation systems do not always achieve adequate indoor air quality. This is generally because natural ventilation conflicts with other requirements for the smooth running of lessons, including thermal comfort and the absence of external ambient noise. In Spain, [33] conducted a monitoring study of 36 schools throughout this country during one school year that reported that the classrooms were in adequate hygrothermal comfort conditions (room temperature and relative humidity) only 68.06% of the time, and they exceeded the set CO_2_ concentration limit during 67.6% of the studied time. Combining both metrics, data showed that the schools were simultaneously within thermally comfortable and adequate indoor air quality parameters only 16.16% of the time. For the Atlantic coast climate, classrooms had acceptable levels of CO_2_ only 27.27% of the time and adequate hygrothermal comfort 74.93% of the time.

In a study in the Basque Country carried out in six schools in the Bajo Nervión (Bilbao) [34] with different outdoor air qualities, all of the studied classrooms exceeded the limit set by the standards of 800 ppm of CO_2_.

In general, the literature recommends air quality improvement systems. In this regard, the Sinphonie guidelines for healthy environments within European schools [35] set out guidelines for improvement in naturally and mechanically ventilated schools based on measurement and live feedback of CO_2_ levels in classrooms. It outlined that classrooms should be equipped with CO_2_ alarms that provide a visual or auditory signal when the level of CO_2_ exceeds a threshold of 700 ppm It also stresses the importance of regular inspection and maintenance of existing mechanical ventilation equipment. 

In the context of the pandemic, the guidance that was available in the beginning of this study in September of 2020 on how to operate schools and educational facilities during the COVID-19 pandemic [36,37,38,39] shared the following general principles:

- Outdoor fresh air supply should be increased.
- Occupancy should be limited in areas where ventilation cannot be substantially increased.
- Air supply filtration with high-efficiency (HEPA) filters should be used.
- Substantially decrease, eliminate or dilute air recirculation in mechanical ventilation systems.

Following these and other similar guidelines (like the University of the Basque Country’s own (presented in Section 1.2), a monitoring campaign was set up during the beginning of the fall term of 2020–2021, when face-to-face teaching was reinstated with the means and methods explained in Section 2.

### 1.2. CO*_2_* Concentration and SARS-CoV-2 Infection Prevention Protocols

CO_2_ is coexhaled with aerosols containing SARS-CoV-2 by COVID-19-infected people and, thus, CO_2_ concentration can easily be used as a proxy of SARS-CoV-2 concentration indoors [40]. In addition to that, it is possible to approximate the quantity of SARS-CoV-2 particulate in a room, provided some conditions are met, which will be discussed in Section 2.3.

Scientific studies confirmed early on that aerosols are one of the main routes for SARS-CoV-2 transmission, so the possibility of airborne transmission increases in high-occupancy indoor environments, such as classrooms. Therefore, international, national and regional protocols and guidelines were established [36,37,38,41,42] with outdoor fresh air ventilation as a requirement for educational buildings since the beginning of the pandemic, while limiting air recirculation [43,44,45].

**Figure 1:**
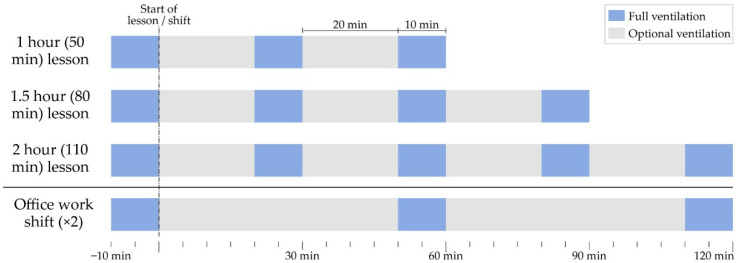
UPV-EHU minimum natural ventilation protocol for classrooms, seminars, laboratories, workshops and office spaces. Source: authors.

In the case of the University of the Basque Country [46], a series of ventilation guidelines were given depending on the type of space. Figure 1 shows the protocol used for classrooms and administration spaces. It was established that minimum continuous ventilation periods of 10 min had to be guaranteed and that all windows and access doors be opened in certain intervals. The scheme was proposed for classes of 50, 80 and 110 min, with at least 10 min of continuous ventilation in every half-hour segment. 

It was the responsibility of the professor or assistant in charge of the lesson to ensure that the ventilation schedule and timing was compliant with the issued protocol. Students could remain in the classroom during the break between classes, but full ventilation had to be performed between lessons. Classrooms, seminars, laboratories, workshops and every other teaching space that did not have any type of natural or mechanical ventilation were excluded from use unless an internal study was carried out to evaluate, specify and approve the conditions for their eventual use.

In the case of offices and administrative spaces, a more spaced-out opening protocol was adopted, with 10 min of window opening every hour.

**Figure 2:**
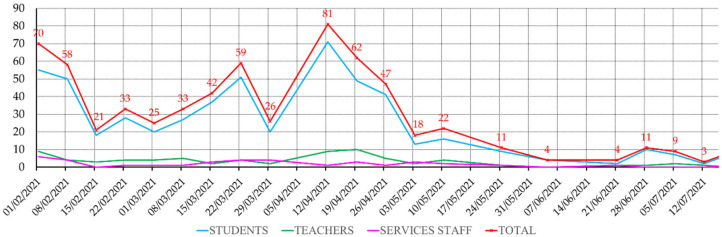
Evolution of the number of cases reported to the different university schools and faculties as registered by the UPV/EHU Surveillance Committee.

In this sense, this study carried out an on-site verification of compliance with this protocol established by the general administration of the University of the Basque Country. The impact of COVID-19 in the period covered by this study can be visualised in Figure 2, as gathered by the University’s own COVID case records. 

**Figure 3:**
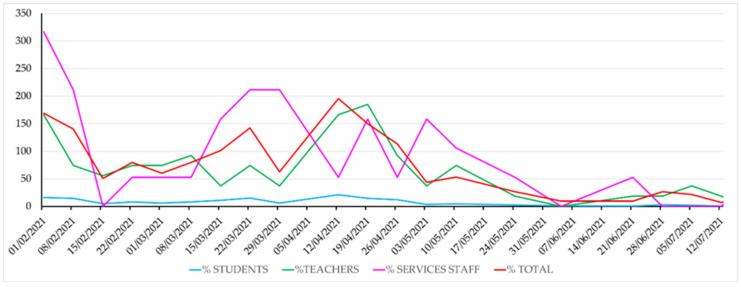
Evolution of the reported SARS-CoV-2 incidence in the studied period by group, in cases per 100,000 people.

Students were logically the most affected group in absolute case number, as they were the most numerous (about 34,000). However, in Figure 3, the virus’s incidence relative to the size of each of the different groups can be seen. Relatively, the most affected were teachers and administrative staff, but this probably has to do with the fact that students did not report the disease to the university’s administration that kept the record, and only their professors were informed that they could not attend classes with mandatory attendance.

## 2. Materials and Methods

The methodology carried out during the project consisted of the development of certain actions and organisation of work, which are described mainly in the following sections. 

### 2.1. Description of the Case Study

**Figure 4:**
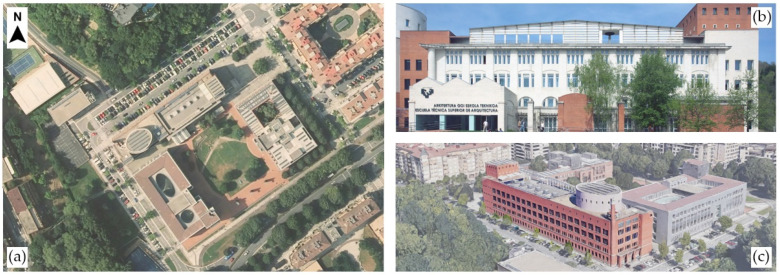
Location of the case study. (**a**) Aerial view. Source: Geoeuskadi. (**b**) Front façade (south), where A 1.1 is located. Source: UPV-EHU. (**c**) Perspective of the north façade oriented to the parking lot. Note that A 2.1 and the Administration space studied are located in this façade. Source: Google Maps.

The analysed spaces are two classrooms and the administration office of the School of Architecture of the UPV/EHU (Figure 4). The current building was inaugurated in 1992 and built with brick facades with an intermediate air chamber. The windows were renovated in various phases in the last decade, and there is not a mechanical ventilation installation except for the basement, auditorium and drawing room on the last floor (former library). 

Two types of university spaces have been studied according to their use. On the one hand, two classrooms with similar floor area but different volume and opposing orientations, both representative of tertiary education classrooms in Spain. On the other hand, the main administration area was monitored This administration area is a small office with a room for 5 people occupied in the regular office hours.

**Figure 5:**
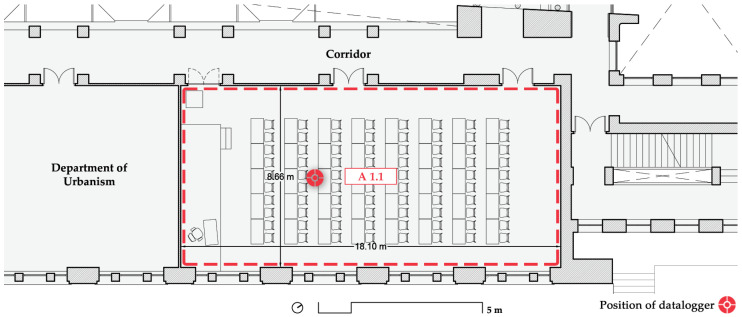
Floorplan of room A 1.1 on the first floor. Source: authors.

Room A 1.1 is a 157 m^2^ classroom with a maximum capacity of 65 students, which was reduced to 50% of that figure due to the pandemic. The height of the classroom is 3.85 m, which gives an approximate volume of 603.50 m^3^. The south-facing windows are big and should allow for a high air change rate when opened. The operation of these windows is manual only and not restricted in any way (professors and students can open or close them at will). The entrance to the room is via a corridor open to a double-height space and thus a considerable volume of air. The corridor does not have much use but can be noisy and, following the University’s protocol, doors had to remain open. However, only two of the three double doors were left open permanently because the computer rack blocks the first one (left in Figure 5). This classroom does not rely on exterior blinds; it can only be darkened by means of interior curtains, and it is mostly used for 1st year lessons of the Bachelor’s Degree in Architecture, with most lessons occurring in the morning and some in the afternoon.

**Figure 6:**
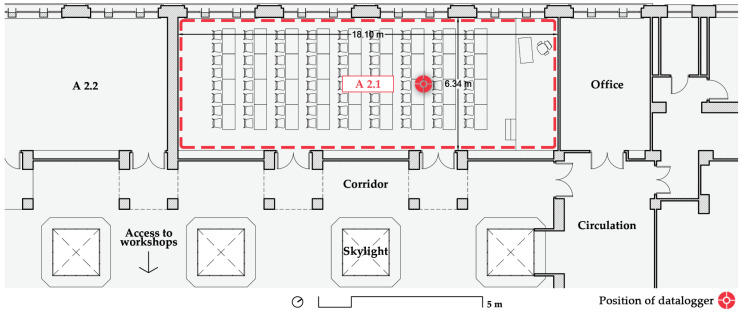
Floorplan of the studied classrooms. A 2.1 on the second floor (top) and A 1.1 on the first floor (bottom). Source: authors.

On the next floor and facing the opposite façade is room A 2.1 (Figure 6), which is a narrower, 115 m^2^ classroom with a maximum capacity of 48 students, which was also reduced by 50%. It is slightly shorter than classroom A 1.1 at 3.11 m, with a volume of 356.90 m^3^. The windows, which have a similar surface area as A 1.1, face the parking lot that can be seen in Figure 4a. This parking lot and the street have a moderate amount of traffic that produces a fair amount of noise. This classroom is devoted to 2nd- and 3rd-year courses, with intensive use through the whole day for theory lessons, as practical lessons are held in the nearby workshops.

**Figure 7:**
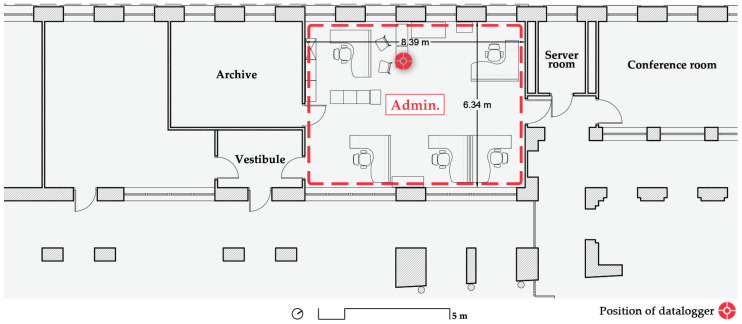
Floorplan of the monitored administration office on the ground floor. Source: authors.

Sharing the same orientation as A 2.1 (Figure 7) and located on the ground floor, the administration office is a 53 m^2^ space where 5 women work on an approximately 8:00–17:00 h schedule with a one-hour lunch break. On Fridays, the schedule is reduced to 8:00–15:00 h without a lunch break. From June through the summer, the regular workday is the same as Fridays. The height of the office is 3.10 m, yielding a volume of 164.90 m^3^. There are two counters or desks facing the hall of the school for servicing students, which can provide some cross-ventilation when open. The only solar protection is provided by interior blinds.

**Figure 8:**
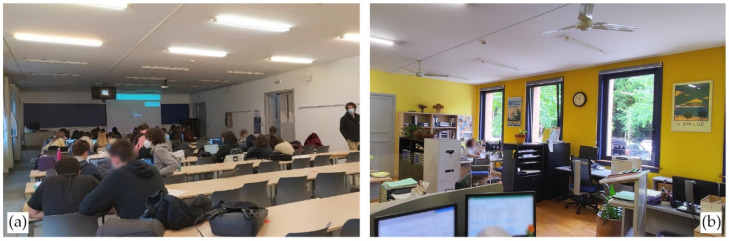
Image (**a**) shows a lesson held in classroom A 1.1 during the winter of 2020–2021, with windows and doors open for cross ventilation. Image (**b**) on the right offers a typical view of the administration office during a workday, with windows partially open. Source: authors.

The characteristics of the described spaces are summarised in Table 1, while Figure 8 provides an idea of them in actual use. Note that despite the fact that the ventilation protocol establishes the aforementioned ventilation schedule, the image shows classroom A 1.1 with full ventilation not only during breaks, but also during lessons. It is also worth mentioning how mandatory mask usage was a measure with very high compliance during lessons, but interpersonal distancing requirements are not met due to the need for groupwork in lessons, despite existing markings on tables to prevent the use of every other seat.

### 2.2. Monitoring and Real-Time Information System

Data showed in this paper were gathered using industrial-grade T&D sensors, which record temperature, relative humidity and CO_2_ concentration. Specifically, the three units used were RTR-576 dataloggers, factory calibrated. The manufacturer declares the following measurement ranges and accuracies:

- Temperature, T: 0 to 55 ± 0.5 °C, with a resolution of 0.1 °C
- Relative Humidity, R.H.: 1 to 95 ± 5% at 50% relative humidity
- CO_2_ concentration, CO_2_: 0 to 9999 ppm ± (50 ppm + 5% of reading)

Recorded data are later funneled through an FTP protocol using the school’s LAN. The procedure is similar to that of [47], where pictures and a diagram can be seen.

For occupant feedback, a display connected to the Internet was set up to display the data in a dashboard form to the students in classrooms A 1.1 and A 2.1. In the case of the administration area, the RTR-576 sensor’s screen itself was deemed to be sufficient to the serve as feedback, due to the reduced size of the room and the familiarity the office workers have with that space.

**Figure 9:**
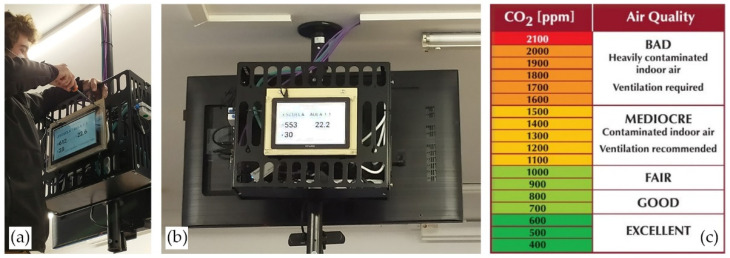
Pictures (**a**,**b**) show the set-up of the displays that provided real-time feedback to the students in rooms A 1.1 and A 2.1, while (**c**) reproduces the legend installed in both classrooms and the administration office. Source: authors.

For occupant feedback, a display connected to the Internet was set up to display the data in a dashboard form to the students in the classrooms, as showed in Figure 9.

For occupants to be able to interpret the data the display was showing them, a legend sticker was placed next to each door and light switch, which was also distributed in a leaflet at the beginning of the 2020–2021 school year. This particular CO_2_ concentration scale is commonly used and was deemed appropriate for this pilot experience in this school as it provided a simple yet fairly detailed classification of CO_2_ levels and is worded in a way that was believed would trigger action by the users. In addition, it has some markings comparing to the regulatory values set forth in RITE [28], which was expected to provoke the occupants to perform some ventilation before reaching the standard’s limit (e.g., users would act when 700 ppm was reached to continue to be on the “Excellent” mark of the chart instead of waiting to surpass the 900 ppm limit for IDA 2).

The data collected were analysed in two ways. First, we analyse the evolution of CO_2_ concentration in the spaces studied throughout the week, during the occupied hours. These data will be used to evaluate the air quality situation with respect to the Spanish RITE [28] regulation. Second, hygrothermal comfort has been analysed according to the international standard ISO 7730:2005 [48].

#### 2.2.1. Framework for Data Analysis

Ventilation requirements have varied in the different regulations in Spain, but always increasing the necessary flow rate. In the RICCACS standard of 1980 [30] and the first RITE of 1998 [49], mechanical ventilation was not mandatory. Since the entry into force of RITE 2007 [28], mechanical ventilation of buildings is mandatory, and its subsequent modifications can be considered minor changes. The ventilation requirements set in this standard depend mainly on the metabolic activity of people, the desired indoor pollutant concentration and the dilution effectiveness (function of the air diffusion system).

A first way to determine the ventilation values is to obtain them from the regulation tables. The minimum ventilation flow rates per occupant and the maximum CO_2_ concentrations (ppm) established in RITE as currently in force and in the new EN 16,798 standard [50] are shown below. The flow rates are practically the same; only in the IAQ category II do they increase from 12.5 to 14 l/s per person. For the CO_2_ concentration values, the limits set by the RITE have been used in the analysis. Table 2 summarises these values:

For thermal comfort, ISO 7730 [48] was used because it is implicit in the CTE building code [51]. The limits were obtained for category II of said standard, with a MET index of 1.1 and a CLO index of 1 during winter. For the summer months, the same MET and a CLO of 0.5 was used, typical for summer indoor clothing. In addition, the thermal comfort limitations established in RITE [28] were observed. This regulation sets the acceptable interval of temperatures for offices in summer at 23–25 °C and the interval for winter at 21–23 °C; relative humidity intervals are set at 45–65% and 40–50% for summer and winter, respectively. In addition, the National Institute of Occupational Safety and Health (INSST) [52] establishes the acceptable range of temperatures in offices with the aim of guaranteeing a percentage of dissatisfaction lower than 10% of 23–26 °C (summer) and 20–24 °C (winter) and a whole year relative humidity acceptance interval of 30–70%.

#### 2.2.2. Phases for Analysis

This study was conducted in several phases in different periods throughout the 2020–2021 and 2021–2022 school years. It should be noted that there may be a large gap between the ventilation protocol of the university and the ventilation pattern actually used in the different rooms, and that this ultimately depended on the users of each space and their changing perception and concern about the importance of ventilation and the significance of avoiding contagions. Therefore, statements about the ventilation actually occurring should be taken as an approximation.

Phase I can be considered an initial deployment of the monitoring equipment and of design of the feedback system described at the beginning of this section. The case studies were monitored but no real time data were provided to the occupants. In the case of classrooms A 1.1 and A 2.1, there was no display installed to show data, and in the administration office, the small display of the datalogger was covered with tape. First results were shared with the head of the School of Architecture internally. For this phase, an analysis of IAQ was performed with the aforementioned methods.

In Phase II, students and teachers were informed that the new displays installed were intended to show real-time CO_2_ levels. A scale of values were explained at which students or teachers should perform more intensive ventilation of the classrooms. Legends with acceptable CO_2_ levels were placed at various points in the classrooms. In the case of the administration area, the information provided by the sensor’s display itself were uncovered. Students, teachers and administrative staff were informed of the acceptable CO_2_ levels, and the janitors were informed or reminded of how to proceed with the opening of the windows. During this phase, winter conditions were evaluated and a week in early May with milder weather outside was also analysed.

Phase III continued into the summer of 2022. Classrooms were empty but the administration office continued work normally. In this case, the aim was to observe whether there had been a change in ventilation patterns and how they affected the comfort of the workspace. In this space, with clearer office hours and constant occupancy, a more detailed study was carried out, taking into account summer comfort.

Table 3 summarises the phases and extent of the analysis for each of the presented rooms:

### 2.3. Model for Retrospective Analysis of SARS-CoV-2

The COVID-19 pandemic has provoked a renewed interest in methods to predict the risk of infection spread [53,54,55,56,57,58], both probabilistic and deterministic, applied to a variety of settings (indoor, outdoor, public transportation [59], etc.), as well as tools [60,61,62] for direct application. The methods and applications differ in the assumptions they make, the data necessary to apply them and in ease-of-use. The classic model of the Wells–Riley equation for determining the likelihood *P* of airborne infection spread in a well-mixed interior space is:

(1) $$P=1-\exp\left(-\frac{I\;p\;q}{Q}\;T\right)\;\;,$$

where *I* is the number of infected people at the beginning of the studied time period *T*; *p* is the ventilation rate of occupants and *q* is the quanta or infectious particle emission rate, both of which depend on activity. The latter also depends on the virus, virus variant, stage of the infection process, etc. 

However, the Wells–Riley equation requires that the ventilation rate *Q* of the analysed spaces be known. This can vary greatly over time, especially in naturally ventilated rooms. We found that Burridge et al. [63] proposed a simple-form equation that allowed us to easily calculate the probability of secondary infection over a certain period using monitored CO_2_ data:

(2) $$P=1-\exp\left(-\int_{0}^{T}\sigma \left(n\right)\;f\frac{I}{n}q\;dt\right)\;\;,$$

where *n* is the number of occupants. Assuming the main factor driving CO_2_ concentration in the studied spaces is human occupancy, as in ordinary classrooms and offices, the fraction of rebreathed air (*f*) is calculated as the difference between the indoor CO_2_ level (*C*) and the baseline outdoor concentration (*C*_0_) divided by the amount of CO_2_ produced in human breathing under the space’s operating conditions (*C_a_*), which gives Equation (3):

(3) $$f=\frac{C-C_{0}}{C_{a}}\;\;.$$

This method was chosen because of its simplicity and because it was specifically developed for both retrospective and predictive analysis, so it allowed us to apply it directly in the monitored spaces where occupancy was constant and known, namely the administration office. This was then applied to said space for phases II and III. 

The selected CO_2_ generation rate per person and occupancy was based on [64] and similar to the original paper [63]. Three different quanta generation rates were used based on [62] and Burridge et al.’s article, as this figure varies widely in the literature [54].

The retrospective analysis was performed on a weekly basis, assuming there is one contagious person (*I* = 1) that could cause secondary infections indivertibly for one work week during the occupied hours. Holidays were logically removed from the analysis.

## 3. Results

This section presents the results obtained in the exploratory analysis carried out during the phases outlined in Section 2.2, according to the methodologies presented in Section 2.

### 3.1. Results for Phases I and II

#### 3.1.1. Phase I

**Figure 10:**
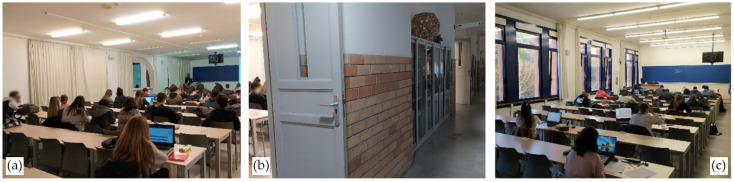
State of the classrooms during lessons in Phase I. (**a**,**b**) show room with door open but ventilation partially restricted to darken for projection and presentation purposes. In picture (**c**) a typical lecture in class A 2.1 with tilted windows can be seen. Source: authors.

From the observation of the spaces and interviews with the students, it was concluded that the most common ventilation protocol in the classrooms was to keep the windows down, some of them completely open, as well as the doors open. This strategy varies from classroom 1.1 to classroom 2.1. In the case of classroom 1.1, priority was given to opening the doors rather than the windows, due to the configuration of the windows and the existence of curtains that prevent natural light from entering through to the interior. Figure 10 shows the interior of a classroom with the windows down or closed and the blackout curtains in front, hindering proper ventilation, and the classroom doors open towards the large hall. 

In the case of classroom 2.1, the use of irregularly tilted windows was prioritised. The classroom shown has a typical pattern of 4th-year students. In the image in Figure 10, a typical occupancy level and half-open windows are observed.

The protocol to be followed with ventilation as established by the University of the Basque Country has not been effectively communicated to students and teachers. The janitors and cleaning services do act at the beginning and end of the day, leaving the windows completely open for approximately one hour.

In the case of the administration, the most senior worker imposed at all times a protocol of permanent opening of the windows in a downcast mode.

Regarding the monitoring of CO_2_ levels, the effect of the availability of information in the classrooms is shown through the study of air quality during three weeks of classes, from 30 November to 20 December 2020 from Monday to Friday. The classes run from 8:00 h to 20:00 h, with breaks from approximately 14:30 to 15:00 h. The analysis of classroom 1.1 is shown, with a natural ventilation capacity somewhat lower than others monitored in which better air quality values have been obtained.

**Figure 11:**
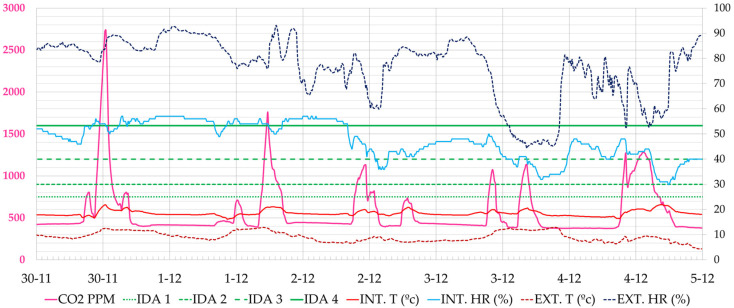
Evolution of CO_2_ concentration, interior and exterior temperature and relative humidity in classroom A 1.1, during the week 30 November 2020 to 5 December 2020 (Phase I). Source: authors.

As an example of the results analysed, the week from 30 November to 4 December 2020 is shown, when the classroom users did not have data. It was a cold week outside (8.0–13.0 °C), and the heating was out of order and working at 30% of its power. Figure 11 shows the CO_2_ concentration values. The maximum values occur at the peak times of morning or afternoon classes. On Monday, 30 December, for example, the highest peak CO_2_ concentration occured at 12:30 h (2738 ppm), on Tuesday at 17:40 h (1759 ppm), on Wednesday at 11:10 h (1132 ppm), on Thursday at 16:20 h (1135 ppm) and on Friday at 13:00 h (1282 ppm). During the week, the air quality remained within the regulated interval (<900 ppm) 75% of the time, marked with the IDA 2 limit. At some moments, high peaks of CO_2_ were reached, the highest values of the four weeks being recorded on Monday, 30 November 2020 with a maximum value of 2738 ppm.

In each monitored week, a count was made of the CO_2_ levels reached, the maximum and minimum levels and the periods in which the classrooms were at acceptable CO_2_ levels. In Section 3.2, these values will be compared with those obtained in Phase II and Phase III. These values are only for the periods of occupation.

**Figure 12:**
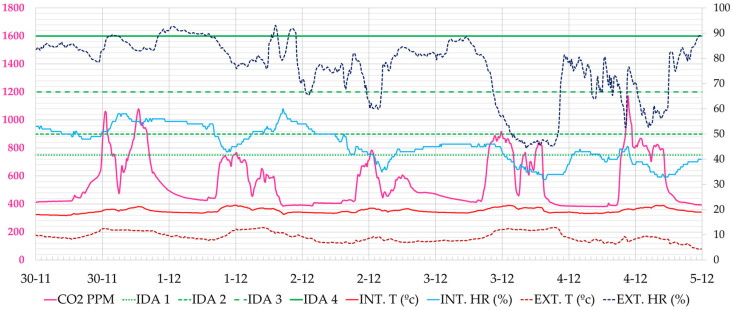
Evolution of CO_2_ concentration, interior and exterior temperature and relative humidity in classroom A 2.1, during the week 30 November 2020 to 5 December 2020 (Phase I). Source: authors.

Similar behaviour can be observed in classroom 2.2, although the lower occupancy together with a more efficient ventilation of the windows, as they are higher and more numerous, provided lower values of CO_2_ concentration with lower intensity peaks. At some moments, high CO_2_ peaks are reached, the highest being the one recorded on Monday, 14 December 2020 with a maximum value of 2181 ppm. Figure 12 shows the typical pattern during this period.

**Figure 13:**
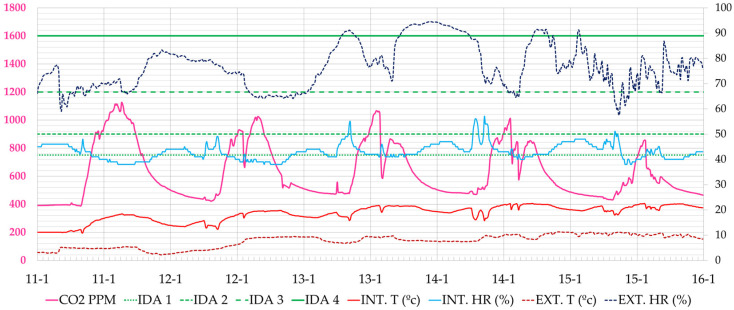
Evolution of CO_2_ concentration, interior and exterior temperature and relative humidity in the administration office, during the week 11–15 January 2021 (Phase I). Source: authors.

As for the administrative area, whose blind monitoring phase began in January 2021, it maintained at all times reduced values of CO_2_ concentration, reaching the maximum value on 11 January 2021 with a maximum value of 1126 ppm. The CO_2_ limit values were reached in short periods during the middle of the day, as seen in Figure 13.

**Figure 14:**
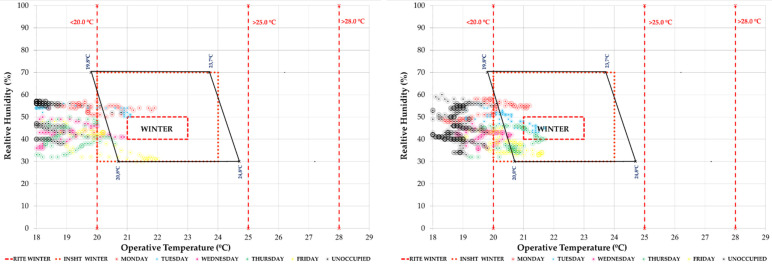
Indoor thermal comfort during the week 30 November 2020 to 5 December 2020 (Phase I) in classroom A 1.1 (**left**) and classroom A 2.1 (**right**). Source: authors.

This study seeks to combine commitment to air quality with maximum comfort. For this reason, the periods of each week within the different comfort categories have been quantified. As an example, the graph corresponding to classroom 1.1 and classroom 2.1 in a typical week of the study period is shown. In Figure 14 we see the temperature/relative humidity pairs of the measurements every 10 min. They have been analysed in relation to three comfort models: the one corresponding to ISO 7730 [48], the RITE [28] and the model of the National Institute for Safety and Health at Work. In this type of graph, the red dashed and dotted lines represent the RITE and INSST comfort limits, respectively, while the polygon outlined in black corresponds to the ISO 7730 thermal comfort model for the conditions defined in Section 2.2.1. It can be seen how it is difficult to enter the comfort zones, more complicated in the cases that have more adjusted models such as the INSST [52] model.

**Figure 15:**
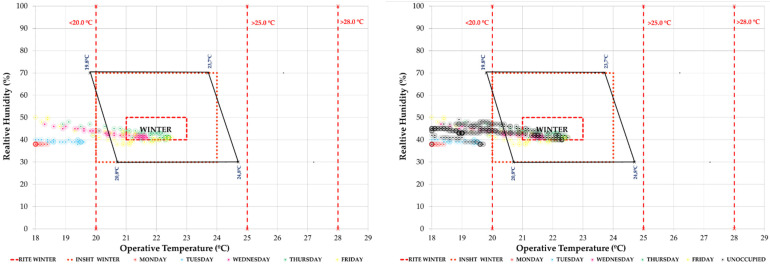
Indoor thermal comfort during the week of 11–15 January 2021 (Phase I) in the administration office. Occupied hours are shown on the (**left**) and all hours, including unoccupied periods and the weekend, can be seen on the (**right**). Source: authors.

As for the administrative area, we observe the effect that the high ventilation has on comfort, making it difficult to reach an adequate temperature during working hours. Figure 15 shows how the unoccupied hours, including Saturdays and Sundays, are in the same comfort range.

#### 3.1.2. Phase II

In this phase, after informing the users of the spaces and indicating the CO_2_ values at which it is convenient to proceed with ventilation, it was observed that in the classrooms there was no apparent commitment to ventilation actions. In the administrative area there was a better adjustment of the ventilation periods, such as closing the windows when the values are very low. In the case of the classrooms, a criterion of permanently lowered windows is maintained.

**Figure 16:**
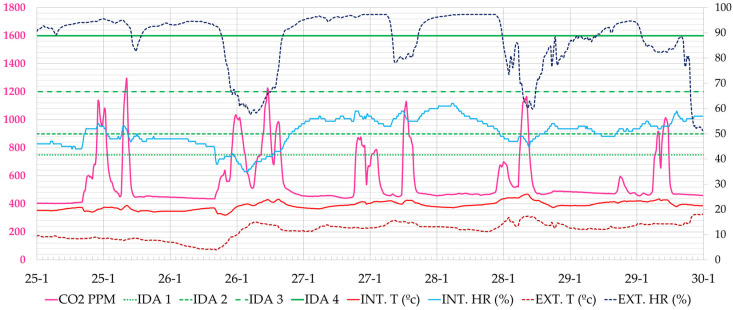
Evolution of CO_2_ concentration, interior and exterior temperature and relative humidity in classroom A 1.1 during the week of 25–31 January 2021 (Phase II). Source: authors.

In this period, we observed that the CO_2_ concentrations in the classrooms did not undergo major changes, obtaining similar average values for the entire period analysed, with a slight decrease in the maximum peaks. In a typical day of this period, the two maximum peaks of CO_2_ in classroom A 1.1 are seen in the morning and in the afternoon, depending on class attendance (Figure 16).

**Figure 17:**
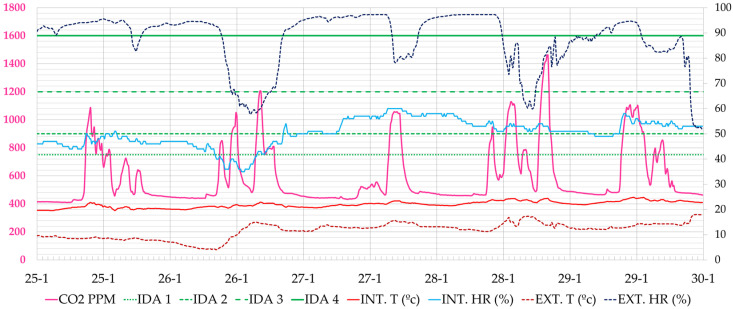
Evolution of CO_2_ concentration, interior and exterior temperature and relative humidity in classroom A 2.1 during the week of 25–31 January 2021 (Phase II). Source: authors.

Classroom 2.1 shows similar records (Figure 17), with the peaks also varying from morning to afternoon depending on the occupancy of the classroom.

**Figure 18:**
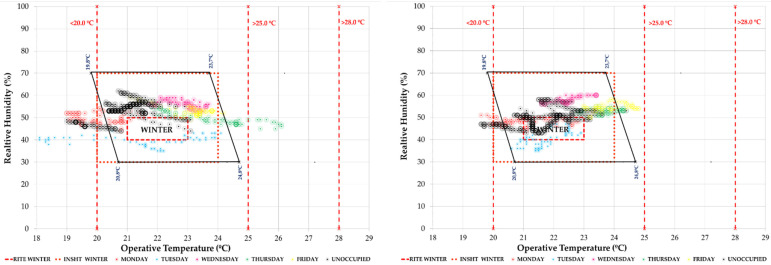
Indoor thermal comfort during the week of 25–31 January 2021 (Phase II) in classroom A 1.1 (**left**) and classroom A 2.1 (**right**). Source: authors.

In terms of hygrothermal comfort, we observe that during this period the classrooms are within the comfort limits of the different standards analysed (Figure 18).

**Figure 19:**
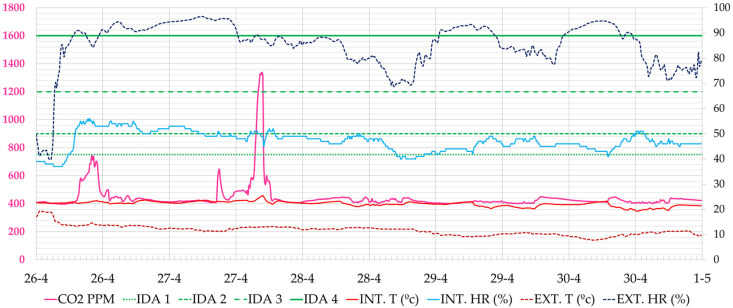
Evolution of CO_2_ concentration, interior and exterior temperature and relative humidity in classroom A 1.1 during the week 26 April 2021 to 2 May 2021 (Phase II). Source: authors.

In addition, a week of April–May 2021 was analysed during which the relatively milder outdoor temperature conditions, although cool during both the day and at night, there was higher solar radiation that affected the indoor temperature. This corresponds in the case of the city of San Sebastian during the periods from September to the end of October and from April to June. The week analysed runs from 26 April to 4 May 2021. In this case, the classroom users had live data through the installed screens. It was a mild week outside (12–17 °C). In this case, the heating was running at full power. In Figure 19, it can be seen that the maximum values of CO_2_ concentration were considerably reduced. On Monday, April 26, there was a peak CO_2_ concentration at 15:40 h (742 ppm), on Tuesday at 16:40 h (1339 ppm), on Wednesday at 13:500 h (448 ppm), on Thursday at 13:50 h (450 ppm) and on Friday at 13:50 h (449 ppm). During the week, air quality remained within the regulation (<900 ppm) 98% of the time, marked with the IDA 2 limit.

The peak observed on Tuesday at 16:40 h, which occurs on Tuesday afternoon during a class with high occupancy and groupwork, is noteworthy. We might deduce that the remote visualisation of the data by the school staff allowed the teacher and students to remain vigilant. It was possible to quickly lower CO_2_ values in less than 10 min by fully opening doors and windows. However, it mut be noted that although similar effects occurred in the other spaces used as case studies during the same phase of the study, real occupancy was uncontrolled in classrooms A 1.1 and A 2.1. 

**Figure 20:**
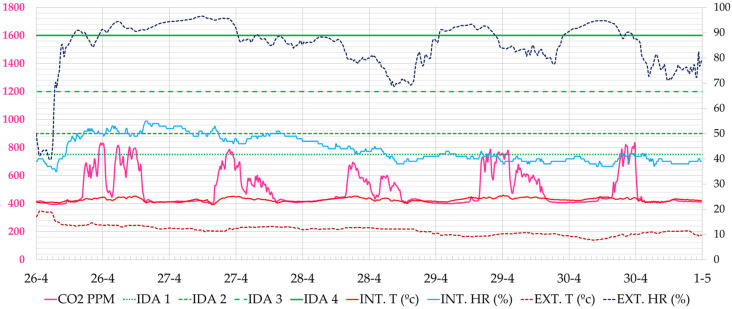
Evolution of CO_2_ concentration, interior and exterior temperature and relative humidity in classroom A 2.1 during the week of 26 April 2021 to 2 May 2021 (Phase II). Source: authors.

In the case of classroom 2.1, it can be seen that the entire time the classroom is kept below 900 ppm CO_2_ concentration (Figure 20).

In terms of thermal comfort, the more precise management of natural ventilation logically allows for greater energy saving by being able to carry out more precise ventilation only at times when the regulatory CO_2_ concentration values are exceeded. However, since strict permanent ventilation protocols are in place, it is difficult to evaluate the overall ventilation system in terms of its energy effects. 

**Figure 21:**
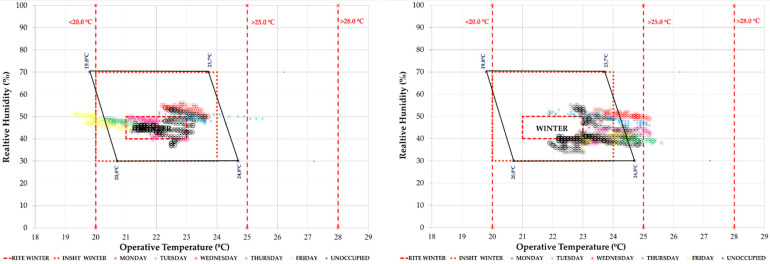
Indoor thermal comfort during the week of 26 April 2021 to 2 May 2021 (Phase II) in classroom A 1.1 (**left**) and classroom A 2.1 (**right**). Source: authors.

In the comparison of both weeks, we see in Figure 21 the comfort graph of T/RH pairs in the week of 26 April. It can be seen how in the school hours from Monday to Friday (in colours), they were mostly within the comfort ranges of the ISO 7730 [48] standard and national regulations (RITE [28] and INSST [52]). The same result is given for unoccupied hours and the weekend (in black). Classroom 2.1 had higher temperature and lower relative humidity values.

#### 3.1.3. Phase III

The purpose of this phase was to evaluate summer comfort with respect to air quality. Since this is a space for administrative use, it is used throughout the year, except during vacation periods. The working hours in this phase are from 8:00 am to 3:00 pm. Between four and five workers work constantly, except for coffee breaks.

**Figure 22:**
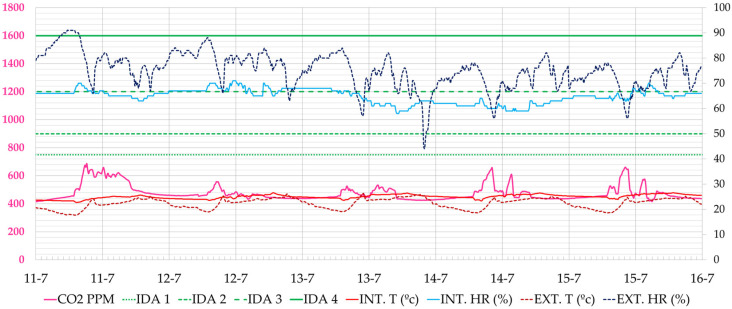
Evolution of CO_2_ concentration, interior and exterior temperature and relative humidity in the administration office during the week of 11–15 July 2022 (Phase III). Source: authors.

Figure 22 shows a typical working day during this period. It can be seen that in this case and with an outdoor climate close to comfort, very low CO_2_ values can be maintained without compromising thermal comfort. CO_2_ levels were below 900 ppm 100% of the time.

**Figure 23:**
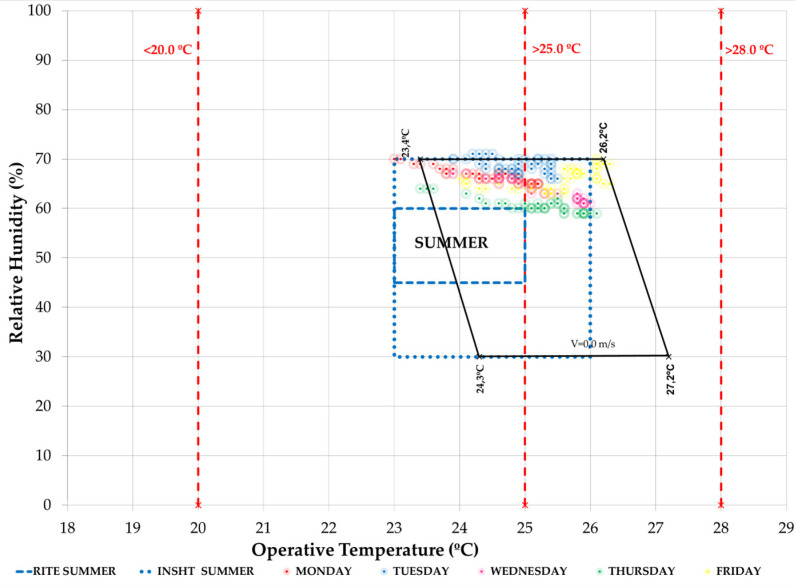
Hygrothermal comfort in the administration office during the week of 11–15 July 2022 (Phase III). Source: authors.

In the hygrothermal comfort analysis model, we see (Figure 23) how the most negative effect that the air flow from the outside can have is that it contributes an excess of ambient humidity, which takes the T/HR pairs out of the more restrictive INSST [52] comfort model.

### 3.2. Comparative Analysis

This subsection offers a comparative evaluation between the different phases of the study. Table 4 and Table 5 summarise the averages of the different phases obtained in the two studied classrooms and the administration office; that is, the CO_2_ levels reached in their maximum, average and minimum and the ranges for air temperature and humidity. The periods in which these values have been located in the different ranges with respect to CO_2_ levels and in the comfort ranges in which the temperature and relative humidity pairs have been located with respect to the indicated national regulations are counted. Likewise, an assessment is made as to whether the analysis phase has registered an improvement in the results with respect to previous phases, using coloured dots. Green and red dots mean improvement and worsening in the performance, respectively, while yellow is used when the difference is deemed not significant or lays inside the error declared for the monitoring devices as presented in Section 2.2.

#### 3.2.1. Classrooms A 1.1 and A 2.1

The comparison is made between Phase I and Phase II-a (winter) and between Phase I and Phase II-b (spring). In general, an improvement in all values is observed, as well as more hours in the comfort zone and better values in CO_2_ levels, especially values below 900 ppm CO_2_. As a summary, the most relevant comparisons are:

- In classroom A 1.1, from 82.8% of the time with CO_2_ levels below 900 ppm in Phase I to 80.6% in Phase II, reaching 98.3% in Phase II-b (spring).
- In classroom A 2.1, from 89.4% of the time with CO2 levels below 900 ppm in Phase I to 83.3% in Phase II-a, reaching 100% in Phase II-b (spring).
- In classroom A 1.1, from less than 1% of the time with thermal comfort within winter limits in Phase I to 21.7% in Phase II-a, reaching 38.3% in Phase II-b (spring).
- In classroom 2.1, from around 7% of the time with a thermal comfort within winter ranges in Phase I to 27.8% in Phase II-a, but only 3.3% in Phase II-b (spring).

Regarding thermal comfort according to the INSHT model:

- Classroom A 1.1 rose from 18.3% of the time with thermal comfort within the winter conditions in Phase I to 78.9% in Phase II-a and more than 91% in Phase II-b (spring).
- In classroom A 2.1, from 42.8% of the time with thermal comfort within winter ranges in Phase I to 88.3% in Phase II-a, dropping to 40% in Phase II-b (spring).

If we combine the CO_2_ limits and comfort values from RITE, the following results are obtained: Classroom A 1.1 goes from 0% of the time within thermal comfort in winter and CO_2_ levels < 900 ppm in Phase I to 19.4% in Phase II-a, reaching 38.3% in Phase II-b (spring). Classroom A 2.1 goes from 5% of the time with thermal comfort within winter ranges and CO_2_ levels < 900 ppm in Phase I to 25% in Phase II-a, but drops to 3.3% in Phase II-b (spring).

#### 3.2.2. Administration Office

The comparison between Phase I (winter without data visualisation) and Phase II (winter with data visualisation) and between Phase I and Phase III (summer with data visualisation) is shown in Table 5. The comparison between winter and summer phases is not very relevant, as the outdoor conditions changed greatly. In general, there is an improvement in all values, more hours in the comfort zone and better values in CO_2_ levels, especially values below 900 ppm of indoor carbon dioxide concentration:

- Data show an improvement from 87.6% of the time with CO_2_ levels below 900 ppm in Phase I to 100% in Phase II, dropping slightly to 98.1% in Phase III (summer).
- There was an evolution from 16.3% of the time within RITE acceptable ranges in comfort for winter in Phase I to 31% in Phase II; however, in Phase III, with different limits for comfort, values within the ranges (summer) were not obtained due to an excess of humidity and temperature.
- A rise from 44.2% of the time within INSST thermal comfort ranges in Phase I to 76.7% in Phase II-a, dropping to 68.6% in Phase III due to the aforementioned reasons.

When CO_2_ concentration and RITE comfort requirement are considered simultaneously in the administration office, values rose from 11.6% of the time with thermal comfort within winter ranges and CO_2_ levels < 900 ppm in Phase I to 41.1% in Phase II-a; however, it dropped to 0% in Phase III due to excess humidity and temperature, as mentioned above.

### 3.3. Retrospective Analysis of SARS-CoV-2

The retrospective analysis of SARS-CoV-2 secondary spread was carried out in the terms explained in Section 2.3 during Phases I, II and III for a total of 10 weeks. This analysis was only performed for the administration office, as with the deployed monitoring campaign it was not possible to determine the occupancy function in classrooms A 1.1 and A 2.1, which renders the model inapplicable.

Equation (3) was used to calculate the fraction of rebreathed air *f* for each of the measurements of CO_2_ concentration. Table 6 shows how *f* varies with CO_2_ concentration considering the typical activity level of an office like the studied space. It compares the *f* values with the recorded absolute minimum, maximum and RITE air quality classification characteristic values.

Equation (2) is used with the 10 min CO_2_ data to calculate the risk of secondary infection on a weekly basis, considering the occupied time only. All ten weeks in Phases I, II and III were analysed using the measurement provided by the RTR-576 sensor and the tolerance declared in Section 2.2. 

**Figure 24:**
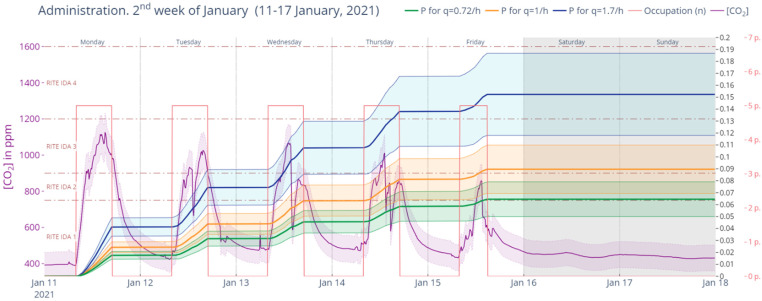
Retrospective analysis of secondary infection risk during the second week of January 2021 (Phase I). Source: authors.

Figure 24 shows the evolution of P using three quanta emission rates (0.72, 1.00 and 1.70 quanta/h) during the second week of January, 2021 (Phase I). This was a week with the highest overall CO_2_ concentration during Phase I in the administration office. There were no holidays. The infection spread risk this week evolved as follows:

- For a 0.72 q/h emission rate, the accumulated P during at the end of the week was 0.0645 or 6.45%.
- For a quanta emission rate of 1 q/h, P was 0.0896 or around 9%
- For q = 1.70 q/h, the total-week secondary spread risk reached 15.23%

The error in the CO_2_ measurement can have a significant effect on the calculation of P, as in the case of this sensor, it is larger the higher the CO_2_ reading is, and in addition, error is accumulated along the analysed week. Burridge et al. [63] noted in their paper that sensors of some precision (±50 ppm) were necessary for the application of the procedure performed in this study. The error is also larger the greater particulate emission rate is.

The analysis, however, allows us to compare between different weeks and the three selected quanta emission rates and to test how large the error might be in a real-life monitored case.

**Figure 25:**
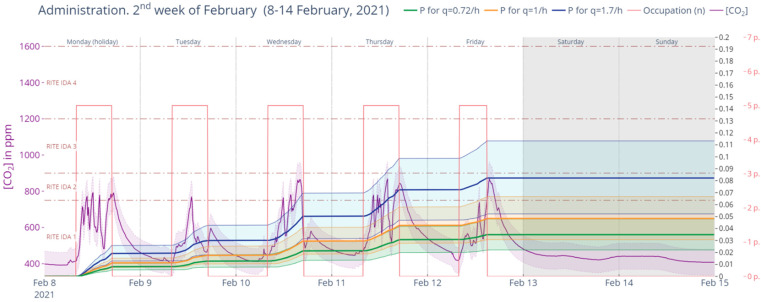
Retrospective analysis of secondary infection risk during the second week of February 2021 (Phase II). Source: authors.

Figure 25 represents the second week of February 2021 in Phase II. This week had uniform indoor CO_2_ levels that resulted in probabilities of 3.49%, 4.84% and 8.23%, respectively, for the aforementioned quanta emission rates.

**Figure 26:**
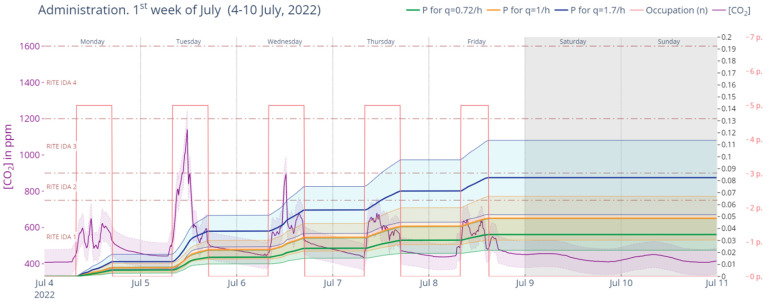
Retrospective analysis of secondary infection risk during the first week of July 2022 (Phase III). Source: authors.

Figure 26 shows the same analysis for the week of 4 to 10 July 2020 during Phase III. This was a week with more moderate overall levels of CO_2_ concentration in the air but a steeper peak in Tuesday 5; this gives probabilities of 3.50%, 4.86% and 8.26%, respectively, for the quanta generation rates of 0.72, 1 and 1.7.

A comparison with the previously analysed week (Figure 25) allows us to see that very different CO_2_ concentration patterns can, however, lead to very similar infection spread probabilities. 

Table 7 summarises the output of the performed analysis in Phases I, II and III.

**Figure 27:**
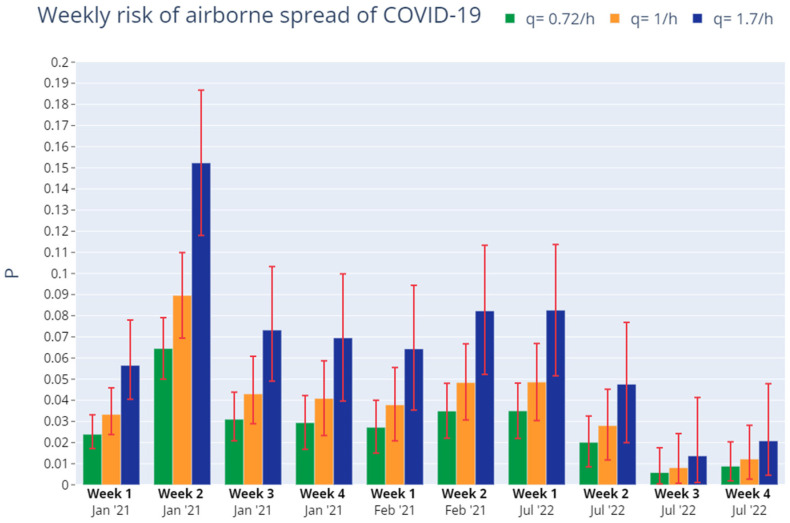
Weekly accumulated risk of secondary airborne spread of SARS-CoV-2 in the administration space in Phases I, II and III. Source: authors.

Figure 27 shows the same results in graphical form. As can be seen, there was no significant reduction in airborne spread risk between Phase I and Phase II except in the case of the second week of January 2021. Also, the analysis for the beginning of July 2022 (Phase III) shows similar risk as Phases I and II. However, a significant decline in airborne spread probability happens in the second to last weeks of July 2022. 

## 4. Conclusions

This pilot experiment was a first step towards the investigation of data-driven and user-informed natural ventilation. As a first step, the real-time information system deployed allowed, to some extent, for the improvement of IAQ and thermal comfort in all the studied spaces in the second phase of the study (both winter and spring) when compared to the blind ventilation protocol provided by the University. In the third phase, which studied the month of July of 2022, there was a severe decline in thermal comfort, as could be expected in an office that does not count on air conditioning during the hot period. However, the application of a simple, easy-to-implement, airborne-virus-spread retrospective analysis method allowed us to check that secondary infection risk did not grow during that same period and instead, declined. 

Despite the limitations derived from the urgent design of the systems that constitute the case study of this paper, the proposed methodology can be further developed and completed to study the alternatives to installation of mechanical ventilation systems in educational buildings that do not currently have one in the Basque Country and Spain. The set-up that was presented in this article could easily be expanded with both commercial and open-source equipment that allow for collection of more data. Window and door opening detection devices would make it possible to evaluate the real window operation patterns and luxmeters would allow us to know how often window closing is related to the use of video projectors and presentations. Other sensors like particulate, dust, CO, and VOC dataloggers would allow a broader IAQ assessment. In addition, some system to control lesson attendance should be added to the classrooms to be able to extend the risk infection model applied to the administration office into the lecture rooms and use it in both predictive and retrospective assessments.

The displays used to provide feedback to users could also be redesigned to show more data or the same data in a more impactful manner, like live calculation of the fraction of rebreathed air, which could trigger disgust feelings in occupants and thus, be more effective at provoking action than the CO_2_ concentration and interpretation legend combination that was deployed.

Further research in the post-pandemic era should, in our view, explore the possibilities of both user-feedback and low-investment actuators or other technologies that take advantage of real-time monitored data to design hybrid ventilation systems in order to obtain a more rational use of natural ventilation and a reduction of energy consumption without having to resort to mechanical ventilation systems, while maintaining or improving indoor air quality with a greater or lesser degree of mechanisation.

In addition, this experiment has allowed, in line with the CBL program of which it is a part, the involvement of the educational community in the air quality of their own study or work center, as well as to increase the training of workers and students at the school of architecture on the regulations, limits and basic operation of indoor air quality in indoor spaces.

## Figures and Tables

**Table 1:** Summary of characteristics of the studied rooms.

	Use	Surface	Volume	Max. Occ.^1^	50% Occ. ^1^	OWA ^2^	OWA/V ^3^
A 1.1	classroom	156.75 m^2^	603.50 m^3^	65	32	6.20 m^2^	0.0102
A 2.1	classroom	114.75 m^2^	356.90 m^3^	48	24	8.20 m^2^	0.0229
Admin.	office	53.20 m^2^	164.90 m^3^	5	-	8.82 m^2^	0.0535

^1^ Occ. is short for occupancy. ^2^ Operable window area. ^3^ Operable window area per unit of interior room volume, in square meters per meter cubed.

**Table 2:** Indoor air quality categories in the referenced standards.

	EN 16798		RITE			This Study
Category	qv (l/s.p)	[CO_2_] Above Baseline	q_v_ (l/s.p)	[CO_2_] Above Baseline		Selected Limit ^1^
				Interval	Default	
Cat. I or IDA 1	20.0	550	20.0	<400	350	750
Cat. II or IDA 2	14.0	800	12.5	400–600	500	900
Category III or IDA 3	8.0	1350	8.0	600–1000	800	1200
Category IV or IDA 4	-	-	-	>1000	1200	1600

^1^ A baseline exterior CO_2_ concentration of 400 ppm was considered.

**Table 3:** Analysis periods and type for the studied rooms.

**Analysis Periods**							
		**A 1.1**		**A 2.1**		**Administration Office**	
		**Start**	**End**	**Start**	**End**	**Start**	**End**
**Phase I**	3 weeks	30 November 2020	27 December 2020	30 November 2020	27 December 2020	4 January 2021	31 January 2021
**Phase II**	(a) 3 weeks	3 January 2021	14 February 2021	3 January 2021	14 February 2021	1 February 2021	14 February 2021
	(b) 1 week	26 April 2021	4 May 2021	26 April 2021	4 May 2021	-	-
**Phase III**	4 weeks	-	-	-	-	4 July 2022	31 July 2022
**Analysis type**							
		**A 1.1**		**A 2.1**		**Administration office**	
**CO_2_ Concentration**		Yes		Yes		Yes	
**Winter Comfort**		Yes		Yes		Yes	
**Spring Comfort**		Yes		Yes		No	
**Summer Comfort**		No		No		Yes	
**Infection Risk**		No		No		Yes	

**Table 4:** Summary of results and phase comparison table for classrooms A 1.1 and A 2.1.

			Classroom A 1.1					Classroom A 2.1				
			Phase I	Phase II (a)		Phase II (b)		Phase I	Phase II (a)		Phase II (b)	
Total analysed hours ^1^			180 h	180 h		60 h		180 h	180 h		60 h	
**[CO_2_]**	Minimum CO_2_ Concentration		386 ppm	403 ppm	•	402 ppm	•	396 ppm	417 ppm	•	406 ppm	•
	Maximum CO_2_ Concentration		2738 ppm	1580 ppm	•	1339 ppm	•	2181 ppm	1457 ppm	•	832 ppm	•
	Average CO_2_ Concentration		614 ppm	674 ppm	•	468 ppm	•	642 ppm	696 ppm	•	581 ppm	•
**RITE Air Quality**	IDA 1	Number of hours	137 h	121 h		59 h		131 h	109 h		53 h	
		% hours	76.1%	67.2%	•	98.3%	•	72.8%	60.6%	•	88.3%	•
	IDA 2	Number of hours	13 h	24 h		0 h		30 h	41 h		7 h	
		% hours	7.2%	13.3%	•	0.0%	•	16.7%	22.7%	•	11.7%	•
	IDA 3	Number of hours	20 h	26 h		1 h		16 h	27 h		0 h	
		% hours	11.1%	14.4%	•	1.7%	•	26.7%	15.0%	•	0.0%	•
	IDA 4	Number of hours	7 h	9 h		1 h		1 h	4 h		0 h	
		% hours	3.9%	5.0%	•	1.7%	•	0.6%	2.2%	•	0.0%	•
**Temperature**	Minimum T.		15.6 °C	17.6 °C	•	19.3 °C	•	16.8 °C	19.6 °C	•	22.0 °C	•
	Maximum T.		22.5 °C	26.1 °C	•	25.5 °C	•	21.8 °C	25.1 °C	•	25.6 °C	•
**Relative Humidity**	Minimum R.H.		30%	33%	•	40%	•	32%	30%	•	37%	•
	Maximum R.H.		74%	61%	•	56%	•	74%	60%	•	55%	•
**Thermal Comfort**	RITE ^2^	Number of hours	1 h	39 h		23 h		12 h	50 h		2 h	
		% hours	0.5%	21.7%	•	38.3%	•	6.7%	27.8%	•	3.3%	•
	INSST ^2^	Number of hours	33 h	142 h		55 h		77 h	159 h		24 h	
		% hours	18.3%	78.9%	•	91.7%	•	42.8%	88.3%	•	40.0%	•
**IAQ Compliance** ^3^	Acceptable	Number of hours	149 h	145 h		59 h		161 h	150 h		60 h	
		% hours	82.8%	80.6%	•	98.3%	•	89.4%	83.3%	•	100.0%	•
	Unacceptable	Number of hours	31 h	35 h		1 h		19 h	30 h		0 h	
		% hours	17.2%	19.4%	•	1.7%	•	10.6%	16.7%	•	0.0%	•
**IEQ Compliance** ^4^	Acceptable	Number of hours	0 h	35 h		23 h		9 h	45 h		2 h	
		% hours	0.0%	19.4%	•	38.3 %	•	5.0%	25.0%	•	3.3%	•
	Unacceptable	Number of hours	180 h	145 h		37 h		171 h	135 h		58 h	
		% hours	100%	80.6%	•	61.7%	•	95.0%	75.0%	•	96.7%	•

^1^ Occupied hours. ^2^ Comfort as defined in Section 2.2.1. ^3^ RITE IDA 1 and IDA 2 only (CO_2_ < 900 ppm) are considered compliant. ^4^ IEQ compliance is evaluated as simultaneous fulfillment of the thermal comfort and IAQ requirement in RITE. Green and red dots mean improvement and worsening in the performance, respectively, while yellow is used when the difference is deemed not significant or lays inside the error declared for the monitoring devices as presented in Section 2.2.

**Table 5:** Summary of results and phase comparison for the administration office.

			Administration				
			Phase I	Phase II		Phase III	
Total analysed hours ^1^			129 h	129 h		105 h	
**[CO_2_]**	Minimum CO_2_ Concentration		397 ppm	411 ppm	•	422 ppm	•
	Maximum CO_2_ Concentration		1126 ppm	869 ppm	•	1141 ppm	•
	Average CO_2_ Concentration		581 ppm	586 ppm	•	536 ppm	•
**RITE Air Quality**	IDA 1	Number of hours	93 h	115 h		100 h	
		% hours	72.1%	89.1%	•	95.2%	•
	IDA 2	Number of hours	20 h	14 h		3 h	
		% hours	15.5%	10.9%	•	2.9%	•
	IDA 3	Number of hours	16 h	0 h		3 h	
		% hours	12.4%	0.0%	•	2.9%	•
	IDA 4	Number of hours	0 h	0 h		0 h	
		% hours	0.0%	0.0%	•	0.0%	•
**Temperature**	Minimum T.		10.9 °C	10.6 °C	•	21.5 °C	•
	Maximum T.		22.9 °C	23.6 °C	•	26.3 °C	•
**Relative Humidity**	Minimum R.H.		31%	36%	•	46%	•
	Maximum R.H.		57%	76%	•	76%	•
**Thermal Comfort**	RITE ^2^	Number of hours	21 h	40 h		0 h	
		% hours	16.3%	31.0%	•	0.0%	•
	INSST ^2^	Number of hours	57 h	99 h		72 h	
		% hours	44.2%	76.7%	•	68.6%	•
**IAQ Compliance** ^3^	Acceptable	Number of hours	113 h	129 h		103 h	
		% hours	87.6%	100.0%	•	98.1%	•
	Unacceptable	Number of hours	16 h	0 h		2 h	•
		% hours	12.4%	0.0%	•	1.9%	
**IEQ Compliance** ^4^	Acceptable	Number of hours	15 h	53 h		0 h	
		% hours	11.6%	41.1%	•	0.0 %	•
	Unacceptable	Number of hours	114 h	76 h		105 h	
		% hours	88.4%	59.9%	•	100.0%	•

^1^ Occupied hours. ^2^ Comfort as defined in Section 2.2.1. ^3^ RITE IDA 1 and IDA 2 only (CO^2^ < 900 ppm) are considered compliant. ^4^ IEQ compliance is evaluated as simultaneous fulfillment of the thermal comfort and IAQ requirement in RITE. Green and red dots mean improvement and worsening in the performance, respectively, while yellow is used when the difference is deemed not significant or lays inside the error declared for the monitoring devices as presented in Section 2.2.

**Table 6:** Relationship between CO_2_ concentration and fraction of rebreathed air in the conditions of the studied spaces.

	CO_2_ Concentration	*f*	*f* (%)
Recorded minimum	386 ppm	0.0000	0.00%
Assumed outdoor baseline	400 ppm	0.0000	0.00%
	600 ppm	0.0053	0.53%
IDA 1 limit	750 ppm	0.0079	0.79%
	800 ppm	0.0092	0.92%
IDA 2 limit	900 ppm	0.0105	1.05%
	1000 ppm	0.0132	1.32%
IDA 3 limit	1200 ppm	0.0158	1.58%
IDA 3 limit	1600 ppm	0.0211	2.11%
	2000 ppm	0.0289	2.89%
Recorded maximum	2738 ppm	0.0615	6.15%

**Table 7:** Summarize the output of the performed analysis in Phases I, II and III.

	Title 2		P for 0.72 q/h		P for 1.00 q/h		P for 1.70 q/h	
**Phase I**	4–10 January	2021	0.0239	+0.0092 −0.0067	0.0333	+0.0126 −0.0095	0.0565	+0.0215 −0.0160
	11–17 January	2021	0.0645	+0.0146 −0.0145	0.0896	+0.0203 −0.0202	0.1523	+0.0345 −0.0343
	18–24 January	2021	0.0310	+0.0128 −0.0102	0.0430	+0.0178 −0.0141	0.0732	+0.0301 −0.0241
**Phase II**	25–31 January	2021	0.0294	+0.0129 −0.0126	0.0409	+0.0178 −0.0176	0.0695	+0.0303 −0.0299
	1–7 February	2021	0.0272	+0.0128 −0.0122	0.0378	+0.0177 −0.0170	0.0643	+0.0301 −0.0289
	8–14 February	2021	0.0349	+0.0131 −0.0128	0.0484	+0.0183 −0.0177	0.0823	+0.0310 −0.0301
**Phase III**	4–10 July	2022	0.0350	+0.0131 −0.0131	0.0486	+0.0183 −0.0182	0.0826	+0.0310 −0.0310
	11–17 July	2022	0.0201	+0.0125 −0.0116	0.0280	+0.0172 −0.0162	0.1523	+0.0293 −0.0272
	18–24 July	2022	0.0058	+0.0117 −0.0053	0.0081	+0.0162 −0.0074	0.0137	+0.0276 −0.0126
	25–31 July	2022	0.0088	+0.0115 −0.0069	0.0122	+0.0160 −0.0095	0.0208	+0.0271 −0.0162

## Data Availability

Data can be made available upon request to the corresponding author.
